# Nusinersen Modulates Proteomics Profiles of Cerebrospinal Fluid in Spinal Muscular Atrophy Type 1 Patients

**DOI:** 10.3390/ijms22094329

**Published:** 2021-04-21

**Authors:** Laura Bianchi, Maria Sframeli, Lorenza Vantaggiato, Gian Luca Vita, Annamaria Ciranni, Francesca Polito, Rosaria Oteri, Eloisa Gitto, Fabrizio Di Giuseppe, Stefania Angelucci, Antonio Versaci, Sonia Messina, Giuseppe Vita, Luca Bini, M’hammed Aguennouz

**Affiliations:** 1 Functional Proteomics Laboratory, Department of Life Sciences, University of Siena, 53100 Siena, Italy; laura.bianchi@unisi.it (L.B.); lorenz.vantaggiato@gmail.com (L.V.); luca.bini@unisi.it (L.B.); 2 Nemo Sud Clinical Centre, 98125 Messina, Italy; mariasframeli@hotmail.it (M.S.); gianlucav.ta81@gmail.com (G.L.V.); 3 Unit of Neurology and Neuromuscular Diseases, Department of Clinical and Experimental Medicine, University of Messina, 98125 Messina, Italy; aciranni@unime.it (A.C.); francesca.polito@unime.it (F.P.); roteri@unime.it (R.O.); smessina@unime.it (S.M.); aguenoz@unime.it (M.A.); 4 Neonatal and Paediatric Intensive Care Unit, Department of Human Pathology in Adult and Developmental Age, University of Messina, 98125 Messina, Italy; egitto@unime.it; 5 Dentistry and Biotechnology, and Proteomics Unit, Centre of Advanced Studies and Technoloy, Department Medical, Oral & Biotechnological Sciences, “G. d’Annunzio”, University of Chieti-Pescara, 66100 Chieti, Italy; f.digiuseppe@unich.it (F.D.G.); stefania.angelucci@unich.it (S.A.); 6 Intensive Care Unit, AOU Policlinico “G. Martino”, 98125 Messina, Italy; anversaci@unime.it

**Keywords:** neuromuscular disease, spinal muscular atrophy type 1, nusinersen, ASO, apolipoprotein A1, apolipoprotein E, transthyretin, haptoglobin, carbonyl groups, oxidized proteins, survival motor neuron (SMN)

## Abstract

Spinal muscular atrophy (SMA) type 1 is a severe infantile autosomal-recessive neuromuscular disorder caused by a survival motor neuron 1 gene (*SMN1*) mutation and characterized by progressive muscle weakness. Without supportive care, SMA type 1 is rapidly fatal. The antisense oligonucleotide nusinersen has recently improved the natural course of this disease. Here, we investigated, with a functional proteomic approach, cerebrospinal fluid (CSF) protein profiles from SMA type 1 patients who underwent nusinersen administration to clarify the biochemical response to the treatment and to monitor disease progression based on therapy. Six months after starting treatment (12 mg/5 mL × four doses of loading regimen administered at days 0, 14, 28, and 63), we observed a generalized reversion trend of the CSF protein pattern from our patient cohort to that of control donors. Notably, a marked up-regulation of apolipoprotein A1 and apolipoprotein E and a consistent variation in transthyretin proteoform occurrence were detected. Since these multifunctional proteins are critically active in biomolecular processes aberrant in SMA, i.e., synaptogenesis and neurite growth, neuronal survival and plasticity, inflammation, and oxidative stress control, their nusinersen induced modulation may support *SMN* improved-expression effects. Hence, these lipoproteins and transthyretin could represent valuable biomarkers to assess patient responsiveness and disease progression.

## 1. Introduction

Spinal muscular atrophy (SMA) is an autosomal recessive disease affecting approximately one in 10,000 live births and having a carrier frequency of about one in 50 [1,2]. This neuromuscular disease is characterized by alpha motor neuron degeneration and progressive muscular atrophy and weakness [3]. On the basis of the clinical severity and time of symptoms onset, four main SMA phenotypes have been identified, ranging from the most severe infantile form: SMA type 1 (SMA type 1; Mendelian Inheritance in Man (MIM) # 253300), to the least severe adult-onset form: SMA type 4 (MIM # 271150). SMA type 1, which constitutes around 60% of SMA cases [1,2], presents symptom onset before the age of six months and it is commonly characterized by severe hypotonia, symmetrical muscle weakness, and respiratory and feeding difficulties. Untreated SMA type 1 patients never acquire the ability to sit, and they usually die within the age of two years because of respiratory failure.

SMA is caused by deletion or, very rarely, by mutations in the Survival Motor Neuron 1 (*SMN1*) gene, located on chromosome 5q13. *SMN1* encodes the homonymous and ubiquitously-expressed SMN protein. Motor neurons present high levels of this protein and they appear particularly susceptible to its defective presence [4]. SMN localises both in the cytoplasm and in the nucleus, where it plays important roles in small nuclear ribonucleoprotein (snRNP) assembly and pre-mRNA splicing [5,6]. Nevertheless, the reason why the lack of SMN protein is responsible for SMA pathogenesis is still unclear.

Different mechanisms have been proposed to explain how aberrant SMN levels may cause at first dysfunctions and then death of motor neurons in SMA. Among these, reduced snRNP assembly, aberrances in transcription and splicing of many genes in brain and spinal cord, and altered transport of β-actin mRNA to motor neuron growth cones [6] are the most debated. In addition, SMN protein has been found to regulate cellular oxidative stress and inflammatory response in microglia [7]. The mechanism for the activation of microglia in SMA pathology is still unknown. However, both SMA patients and model mice show astrogliosis in the spinal cord and inflammatory cytokines are released from activated astrocytes [7,8].

The complete loss of the SMN protein is embryonic lethal [9] but in humans, the presence of a Survival Motor Neuron 2 (*SMN2*) gene, which provides a small quantity of functional SMN protein, allows for embryonic and fetal development [10,11].

The *SMN2* gene differs from *SMN1* by five nucleotides, but the only 840C→T single nucleotide transition, at exon 6–7, is crucial. This disrupts the binding site for splicing modifiers and determines the exclusion of exon 7 from the overwhelming majority of *SMN2* transcripts [12]. The result is a truncated and not completely functional protein that is rapidly degraded. Despite the fact that only 5–10% of the full-length SMN is produced by the *SMN2* gene, the clinical severity of SMA is related to the number of *SMN2* copies: patients with a higher copy number generally show a milder phenotype [13,14]. Accordingly, the *SMN2* gene was suggested as a possible therapeutic target and different curative approaches have been focused on modifying the *SMN2* pre-mRNA splicing [15].

The course of SMA is invariably progressive. In spite of this, the recent advent of new therapies, such us the antisense oligonucleotide (ASO) nusinersen, has modified the natural history of this disorder. In cellular and animal models, it acts by promoting the inclusion of the exon 7 in the *SMN2* mRNA and by increasing the intracellular levels of functional protein produced from this gene, with no correlation with cerebrospinal fluid (CSF) SMN protein levels [16,17]. Clinical trials and post marketing studies proved the intrathecal nusinersen administration inducing an SMN protein increase in motor neurons of exposed infants and a favorable benefit-risk profile [18,19,20,21]. In particular, the 51% of SMA type 1 probands, who received treatment during the first seven months of life, achieved new motor-milestones and therapy increased the overall survival rate and the proportion of infants that did not require permanent assisted ventilation [19].

On the other hand, the availability of new drugs in SMA management has also increased the necessity to clarify the biochemical response they elicit and to define how the treatment acts in ameliorating patients’ life expectancy [22,23,24]. Proteomics is a valid ally in this challenge: it may in fact contribute to delineating the molecular bases of the disorder and in identifying biomarkers to evaluate and predict responsiveness to the treatment. Recently, a number of investigations has in fact proved the reliability in applying proteomics to understand SMA pathogenesis for biomarker finding and novel pharmacological attempts [25,26,27,28]. Here, we applied two-dimensional SDS-polyacrylamide gel electrophoresis (2D-PAGE), MALDI-TOF mass spectrometry (MS), and 1D and 2D western blot (wb) to assess the biochemical response to nusinersen administration in 10 SMA type 1 patients after six months of therapy. All patients received 12 mg/5 mL × four doses of loading regimen administered at days 0, 14, 28, and 63, as recommended.

As CSF composition is strongly influenced by neuron and glial cells metabolism and physiology, the differences we detected among CSF proteome profiles before and after the treatment may be considered possible biomarkers of an early neuronal response to nusinersen. In particular, we proved the therapy inducing several significant variations in proteoform patterns of apolipoprotein A1 (APOA1) and transthyretin (TTR) and in the overall abundance of apolipoprotein E (APOE). As these proteins are active in several processes previously reported affected in SMA, their functions, directly or indirectly influenced by nusinersen, may contribute towards improving the disease outcome.

Our work is the first proteomics investigation showing nusinersen effects in CSF from SMA type 1 patients and, despite further analyses are needed, obtained results may represent a precious step forward in monitoring the course of the pathology for more accurate prognosis and patient care.

## 2. Results

### 2.1. Clinical Data

Ten SMA type 1 patients, eight male and two female, have been enrolled in the study, and their ages ranged between 2 and 28 months (mean: 9.5; SD: 10.02). Table 1 shows patients’ clinical data and their classification according to the Dubowitz decimal system [29,30] and the more recent “ABC” SMA type 1 classification [31]. After the four loading doses of nusinersen, administrated in the first two months, all patients were alive et 180 days, none showed adverse events, and they all had a significant amelioration of motor functions (Table 1) assessed by the CHOP INTEND scale, with none showing any negative change.

At baseline the CHOP INTEND scores of the patients ranged between 9 and 43 (mean = 26.9; SD = 11.37). Six months after starting therapy, patients significantly improved (*p* = 0.00512, Wilcoxon paired test), ranging between 35 and 48 (mean = 40.1; SD = 6.19), with an increase between +3 and +26 (mean: 12.2; SD: 6.51). Patient (pt.) 3, who had already achieved a good head control before the treatment, maintained this skill, and further 3 patients, pts 4, 5, and 7, acquired the ability to maintain the head upright.

Despite the improvement of motor functions observed in pt. 1, he had several respiratory infections after which he showed a worsening of breathing and required longer use of non-invasive ventilation and he also needed the nasogastric tube for nutritional support (Table 1). Similar clinical course had the pt. 10, who had a pneumonia after the second dose of nusinersen. At baseline, he had already needed enteral nutritional support by orogastric tube and after the first injection he underwent percutaneous endoscopic gastrostomy (PEG) tube insertion.

### 2.2. Cerebrospinal Fluid Proteomics Profiles

Proteomic profiles of cerebrospinal fluid (CSF) specimens at baseline (T0 = immediately prior to the first nusinersen administration) and 6 months after the treatment initiation (T1 = immediately prior to the fifth drug administration) were delineated for pts 1–7 (Table 1). CSF samples from seven age-matched non-SMA individuals (four male and three female, age range: 1–36 months), subjected to lumbar puncture for diagnostic reasons, were applied to obtain control proteomic profiles (Ctrls 1–7). CSF samples from pts 8–10 and Ctrls 8–10 were instead used for western blot (wb) analyses. We decided to validate image-analysis/statistics results by principally applying samples excluded from the gel analysis in order to increase the reliability of the obtained 2D-PAGE/MS data. In addition, due to the consistent amount of protein load required for the classical proteomic approach we applied, we did not dispose of sufficient protein samples from pts 1–7 and Crtls 1–7 to perform, for each one of them, further 2D-PAGE analyses.

**Figure 1:**
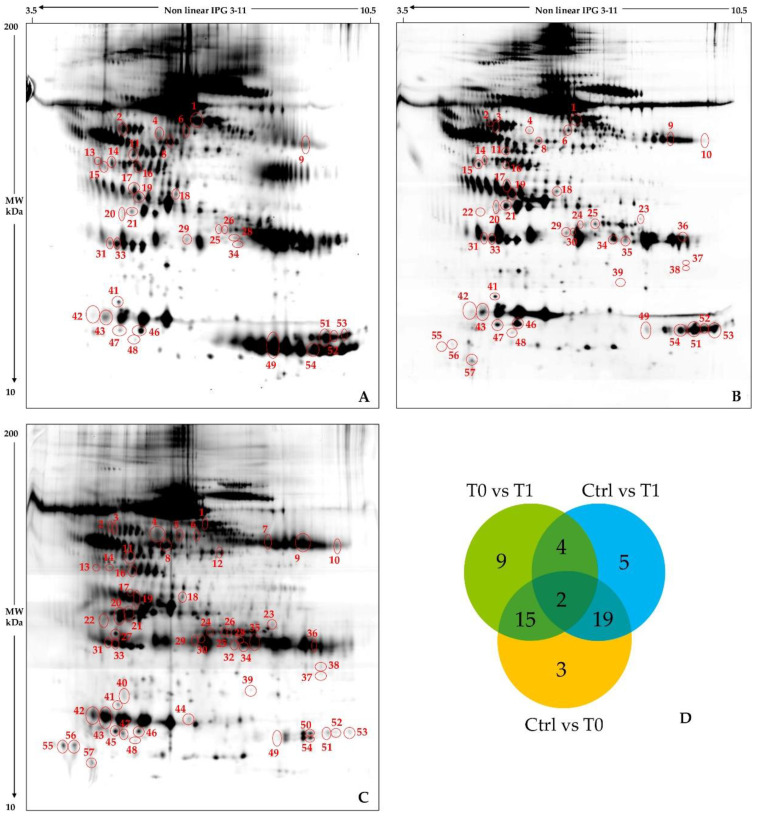
CSF protein patterns of SMA type 1 patients at baseline and after six months of nusinersen therapy and control subjects. Silver stained reference 2D gels of CSF samples from: SMA type 1 pt. 5 before nusinersen treatment (**A**), the same SMA type 1 patient after six months of nusinersen therapy (**B**), and control subject Ctrl 4, who underwent a lumbar puncture for diagnostic reasons (**C**). Red circles and numbers point out differentially abundant protein spots detected by comparing patients’ and control groups: T0 vs. T1, Ctrl vs. T0, and Ctrl vs. T1. Numbers correspond to those listed in Table 2 and in Supplementary Table S1. The Venn diagram (**D**) shows the number of the differentially abundant proteins we detected and it allowed an overall evaluation on their distribution among the three different performed comparisons.

Gel-image analysis, rigorous statistics, and result filtering for % Vol ratios ≥2 allowed detecting highly significant protein-differences: 30 between T0 and T1 sample classes, 39 between control and T0 groups, and 30 between control and T1 classes, Supplementary Table S1. Representative 2D gel images of the three analysed classes are shown in Figure 1 (A. ET0, B. ET1, and C. Ctrl 4). Here, detected differences are evidenced by circles and numbered as listed in Supplementary Table S1. Several proteins of interest were shared (i) by the Ctrl vs. T0 and the Ctrl vs. T1 comparisons (19 spots), and (ii) by the Ctrl vs. T0 and T0 vs. T1 comparisons (15 spots), as highlighted by the Venn diagram in Figure 1D. Of great relevance, 13 out of the 15 (ii) shared differences are qualitative differences being corresponding spots not detected in the T0 class. Conversely, mean relative abundances of these differing spots are very similar between the T1 and the control groups. This suggests a positive effect of nusinersen in reducing dysregulation of specific CSF proteins, which may be applied as markers of early responsiveness to the treatment. On the other hand, among the (i) 20 differing proteins, nine spots were not detected in either T0 or T1, 2 spots were not observed in control samples, and the majority of the other nine quantitative differences did not present abundance improvements when compared to the control group. Further studies are needed to increase the generalizability of these findings and to clarify whether more prolonged treatments may further change protein abundance/occurrence in CSF of SMA type 1 probands.

### 2.3. Principal Component Analysis and Hierarchical Clustering

**Figure 2:**
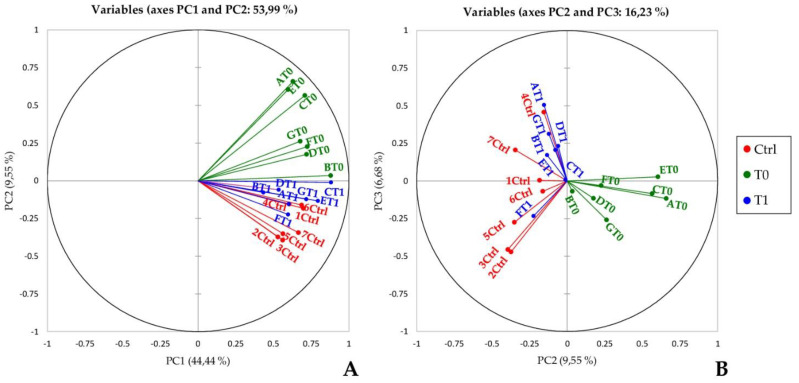
Principal Component Analysis. Principal Component Analysis (PCA) performed on relative % Vols of spots matched among T0, T1, and control samples. Graphs highlight spatial distribution of the 21 CSF analyzed samples [14 from SMA type 1 pts 1–7: 7 before (T0) and 7 after (T1) six months of nusinersen treatment; and 7 from control subjects] along the PC1 and PC2 (**A**) and along the PC2 and PC3 (**B**). T0 samples are in green, T1 in blue, and Ctrl in red.

By taking advantage of XLStat-OMICs, a principal components analysis (PCA) of all the spot % Vol values detected in each gel was performed to evaluate the variance-covariance occurring among protein patterns from the three tested conditions. The principal components PC1 vs. PC2 plotting, which accounts for the 53.99% of total variance, reveled an evident shift of treated probands toward controls. Vectors representing gels from controls and treated patients had in fact similar orientation and corresponding bubbles clustered closely (Figure 2A: red and blue vectors/bubbles, respectively). PCA also highlighted, both at baseline and after the nusinersen administration, that the variance is subject-dependent. While PC2 mainly accounted for the spreading of SMA type 1 patient proteomic-profiles at T0 (Figure 2A: green bubbles), PC1 principally defined the range of the treated-patient cluster (Figure 2A: blue bubbles), thus bona fide reflecting individual-dependent response to the therapy. In the PCA plot with PC2 vs. PC3, which represents the 16.23% of total variance, control/T1 samples, which clustered in PC1 vs. PC2 plot, stretch throughout the PC3 without a clear separation, considering the PC3, between controls and T0 samples (Figure 2B).

**Figure 3:**
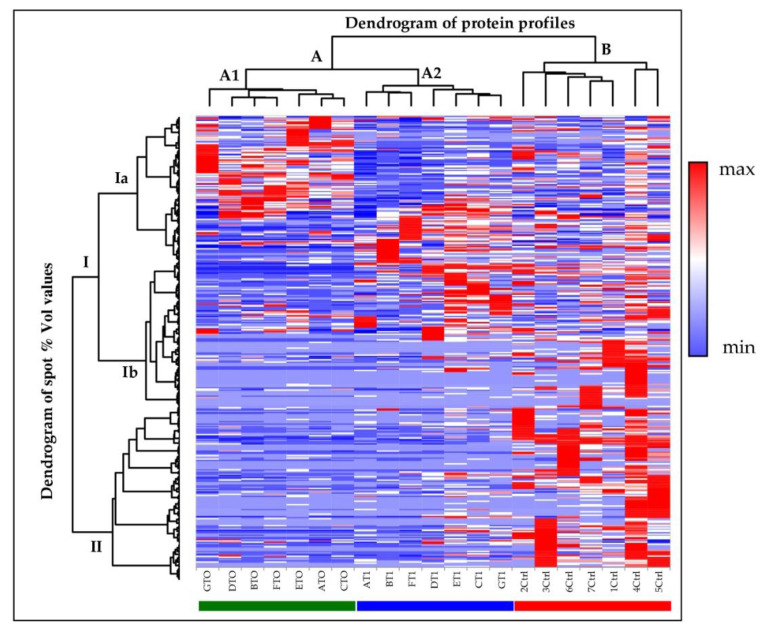
Heatmap Analysis. Heatmap analysis performed on matching spots among the three conditions (T0, T1, and Ctrl). Color change blue to red indicating less or higher protein abundance, respectively. Each row corresponds to a protein spot while each column corresponds to individual sample gel. The dendrogram of spot % Vol values (on the left of the heatmap) highlights protein clustering according to protein abundance similarity (three main protein groups were delineated: Ia, Ib and II). The dendrogram of protein profiles (on the top of the heatmap) evidences three main gel clusters: they correspond to T0 (cluster A1), T1 (cluster A2), and Ctrl (cluster B) samples.

XLStat was also applied for graphically representing all spot % Vol values across the investigated samples. The generated heatmap allowed an immediate evaluation on protein abundance profiles characterizing each subject, and clusters of spots with similar or vastly different % Vol values were clearly visible (Figure 3). Although at different hierarchical levels, spots were grouped into three main clusters (i.e., Ia, Ib, and II), as evidenced by the “dendrogram of spot % Vol values” on the left of the expression matrix. Despite individual variability, the general spot abundance-trends from analysed subjects clustered, by similarity, into three groups (i.e., A1, A2, and B) that exactly corresponded to the T0, T1, and control sample-groups, as also stressed by the “dendrogram of protein profiles” on the top of the matrix. Notably, the T1 (A2) cluster was interposed between the T0 (A1) and the control (B) clusters. This suggested nusinersen administration had modified the proteomic pattern of treated patients bringing them closer to those of the controls. Nevertheless, the protein profiles of the T1 CSF showed more similarities with the T0 CSF than with the control ones, as even evidenced by the different hierarchical level of the T0 (A1) and T1 (A2) clusters and of the control (B) one.

Of note, the three main clusters from the dendrogram of spot % Vol values and the three clusters from the dendrogram of protein profiles intersected in the expression matrix and clearly evidenced three main areas of differential expression among T0, T1, and control groups. A generalized inversion of protein abundance distinguished controls from SMA type 1 patients before treatment. While the Ia cluster roughly grouped protein spots up-regulated in T0 and down-regulated in controls, Ib and II clusters grouped protein spots presenting low % Vol values in T0 specimens and medium-low (Ib) or high (II) abundance in control CSF. Post-treatment collected CSF instead showed an up-regulation of protein spots mainly from the cluster Ib and in part from the cluster Ia, and a down-regulation of the majority of Ia clustered spots, part of the Ib spots, and the entire II cluster.

### 2.4. REVIGO Analysis on Biological Process GO-Terms Annotating Identified Proteins of Interest

**Figure 4:**
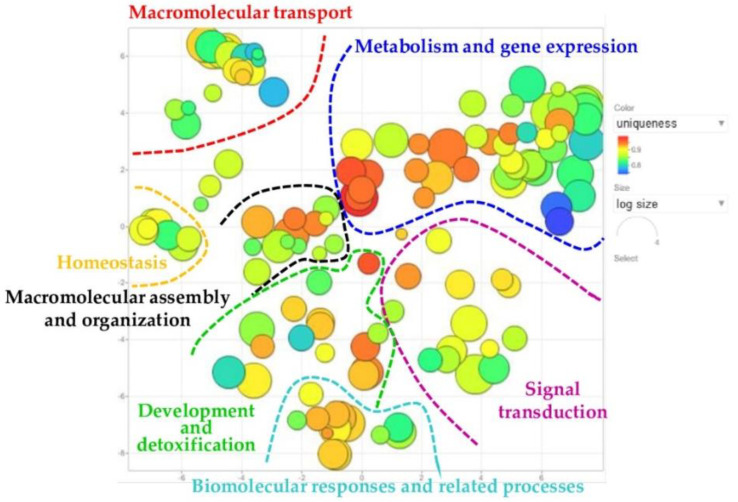
REVIGO scatterplot of Biological Process GO-terms annotating the significantly differing proteins identified between T0, T1, and Control samples. REVIGO scatterplot showing semantic similarities among Biological Process GO-terms, from identified differences, clustered in a 2D space by multidimensional scaling. Bubble radius indicates the frequency of individual GO terms in the GO annotation (GOA) referring database (SwissProt): bubbles corresponding to more general terms (higher in hierarchy) are larger than those representing more specific terms (lower in hierarchy). Bubble color indicates the GO annotation uniqueness in the GO list: from brilliant red, for common shared terms, to dark blue for unique individual terms (with lower *p*-values). Semantically similar GO terms are clustered and outlined by dashed colored lines. Summarized GO terms are listed in Supplementary Table S2.

MALDI-TOF MS was then applied to identify significantly differing protein spots. Thirty of them were detected in MS preparative gels, excised, and analysed. Seventeen protein spots, corresponding to six unique proteins, were hence unambiguously identified (Table 2). The biological meaning of the differentially abundant proteins was at first investigated by a general overview on Gene Ontology (GO) terms annotating the 6 proteins of interest in UniProtKB. Redundancy of Biological process (BP) GO terms was reduced and summarized terms were visualized by applying REVIGO resource, as shown in Figure 4. Here, GO terms were clustered according to semantic similarities and regardless of their corresponding proteins.

Seven main semantic-groups, highlighted in the plot by curved lines, were delineated: (i) metabolism and gene expression, (ii) macromolecular transport, (iii) development and detoxification, (iv) signal transduction, (v) biomolecular responses and related processes, (vi) macromolecular assembly and organization, and (vii) homeostasis. The numbering order, (i)–(vii), was established according to the cluster size, from largest to smallest, and each cluster name was arbitrarily assigned based on the preponderant biological function specified by the corresponding clustered GO terms.

In Supplementary Table S2, the GO terms summarized by REVIGO were listed according to their distribution into the delineated term groups. The 19 GO terms with neurological implications were highlighted in bold and numbered, as well as their corresponding bubbles in Supplementary Figure S1. They spanned throughout the seven functional clusters and described the proteins of interest involved in: neuron projection and dendritic spine development (cluster iii) and maintenance (cluster vi), peripheral nervous system axon regeneration (cluster v), cholinergic synaptic transmission (cluster iv) and synaptic organization (cluster vii), long-term potentiation, and plasticity (cluster vi), clustering of N-methyl-d-aspartate (NMDA) type of glutamate receptor for excitatory transmission (cluster ii), long term memory (cluster iii) and regulation of behavioral fear response (cluster v), regulation of neurofibrillary tangle assembly (cluster vi), amyloid precursor metabolism (cluster i) and clearance (cluster iii), and neuron death (cluster i).

### 2.5. Western Blot Profiles of APOE, APOA1 and Transthyretin in CSF Samples from SMA Type 1 Patients (T0 and T1) and Control Subjects

**Figure 5:**
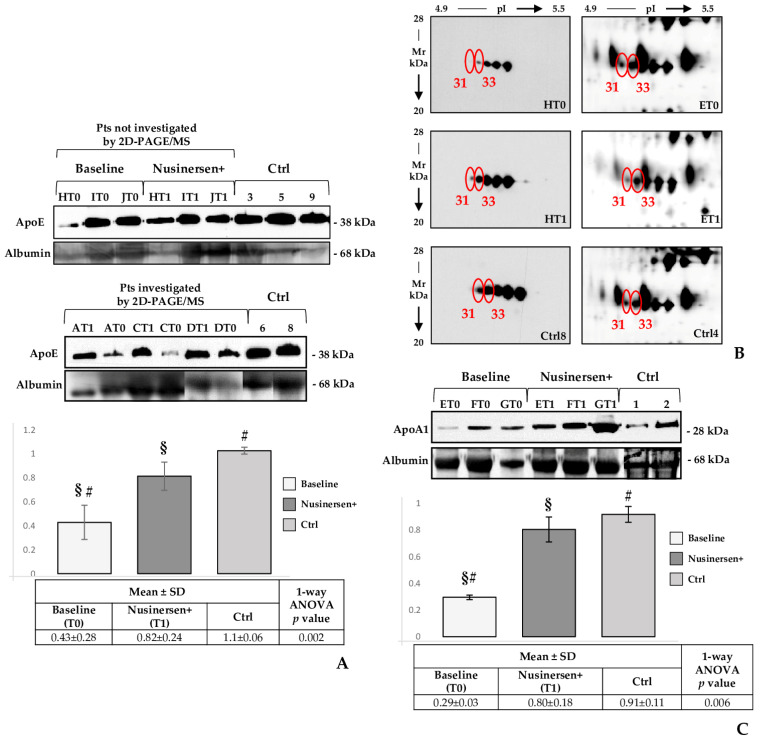
APOE monodimensional and APOA1 two-dimensional western blot. (**A**). Immunostained APOE signals on CSF samples from pts not investigated by 2D-PAGE/MS (pts 8–10 = HT0/1, IT0/1, and JT0/1 samples), pts investigated by 2D-PAGE/MS (pts 1, 3, and 4 = AT0/1, CT0/1, and DT0/1 samples), and Ctrl subjects investigated and not investigated by 2D-PAGE/MS (Ctrl 3, 5, and 6; Ctrl 8 and 9, respectively). The histogram shows the mean of normalized relative-integrated-density ± SD values of T0 (baseline, *n* = 6), T1 (after six months of nusinersen therapy = nusinersen+, *n* = 6) and Ctrl (Ctrl, *n* = 5). A significant increase was detected among T0 and T1 samples and among T0 and Ctrl samples. **#** and **§** indicate significant abundance changes (*p* = 0.002) occurring between controls and T0 patients, and between T0 and T1 probands, respectively. Analysed patients and controls are reported on the top of the corresponding wb band. (**B**). on the left: CSF samples from pt. 8, before (T0) and after (T1) nusinersen therapy, and from Ctrl 8 were resolved by 2D SDS-PAGE and analysed by western blotting using an anti-apolipoprotein A1 monoclonal antibody. The 2DE wb evidenced a substantial increase in APOA1 signals induced by the treatment. Wb signals corresponding to identified APOA1 spot-differences are highlighted by red circles. Numbers corresponded to those reported in Figure 1 and Table 2, as well as in the enlargements of silver stained gels, from ET0, ET1, and Ctrl 4 samples, here shown: (**B**). on the right. (**C**). Immunostained APOA1 signals on CSF samples from three SMA type 1 patients (pts 5–7), previously investigated by 2D-PAGE/MS, at T0 (ET0, FT0, and GT0; baseline) and T1 (ET1, FT1, and GT1; after six months of nusinersen therapy = nusinersen+) and from previously investigated control subjects (Ctrls 1 and 2). The histogram shows the mean of normalized relative-integrated-density ± SD values of T0 (*n* = 3), T1 (*n* = 3) and Ctrl (*n* = 2). A significant increase was detected among T0 and T1 samples and among T0 and Ctrl samples. **#** and **§** indicate significant abundance changes (*p* = 0.006) occurring between controls and T0 patients, and between T0 and T1 probands, respectively.

The two APOE protein spots (*n*. 17 and 19, Figure 1 and Table 2) we identified significantly differed among probands’ CSF and control samples. On the contrary, they showed very similar abundance levels in patients both before and after nusinersen administration. This differential occurrence of different proteoforms among T0/T1 and the control subjects may be correlated to APOE differential functionalities. With the intent to report on its abundance difference, as suggested by the 2D-PAGE/MS analysis, between SMA probands and control children, we tested by 1D wb APOE in pts 8–10 and Ctrl 3, 5, and 9 (Figure 5A—pts not investigated by 2D-PAGE/MS). Although we cannot advance any hypothesis regarding a therapy-dependent functional modulation of APOE proteoforms, wb evidenced that the nusinersen treatment led to a significant APOE up-regulation, with T1 intensity values getting closer to those from controls than from T0 samples (Supplementary Figure S2). Reasonably, other APOE proteoforms, which we did not identify by MS, determined the different wb signal intensity of this lipoprotein among patients at baseline and after the treatment. Nonetheless, as the data from 1D wb of pts 8/10 is not in line with the abundances of the two identified APOE spots in pts 1–7, we performed 1D wb in T0 and T1 specimens from three patients (pts 1, 3, and 4, for which we still had some sample, although not sufficient for a 2D wb) analyzed in 2D-PAGE/MS. Statistical analysis was perform considering all the values obtained from the six T0, six T1, and five Ctrl samples investigated by wb (Figure 5A, histogram and table).

A therapy-related improvement of the two identified APOA1 proteoforms (n. 31 and 33, Figure 1 and Table 2) was demonstrated by 2D-gel image-analysis, although only spot 33 resulted significantly more abundant in T1 than in T0 samples. We indeed validated this result by 2D wb. As shown in Figure 5B (on the left), nusinersen induced in pt. 8 (HT0 and HT1 samples) an evident increase of APOA1 signals and the T1 APOA1-profile was practically identical to that of the control sample. On the left side of Figure 5B we reported enlargements from silver stained reference gels, shown in Figure 1, for properly correlating MS identified APOA1 spots with wb signals of this lipoprotein. Although the APOA1 results were in line with 2D-PAGE/MS data, we investigated APOA1 abundance in 1D wb to improve the relevance of acquired data. Three patients (pts 5–7, T0/T1) and two controls (Ctrl 1 and 2) were analysed among those applied for 2D gel-image analysis and MS, for which we disposed of some sample but not sufficient for a 2D wb. As they localized at the same Mw, all the APOA1 2D wb signals were retained resolving in the same 1D band. Indeed, obtained results from 1D wb are indicative (Figure 5C) of a significant generalized increase of APOA1 abundance in consequence of nusinersen administration.

Transthyretin (TTR) is the most representative protein among the protein spots we identified (spots n. 20, 43, 46, and 48, Figure 1 and Table 2). According to our data, nusinersen therapy induced an evident up-regulation of all the identified isoforms. While the % Vol values of spots 20 and 43 increased, if compared to baseline T0, with a restoring trend toward the corresponding control values, spots 46 and 48 presented a significant abundance increase in T1 even when compared with the control group. In order to confirm these data, central nervous system (CNS) samples from pt. 9, before (IT0) and after (IT1) the ASO treatment, were analyzed by applying 2D wb and immunostaining with an anti-TTR Ab.

**Figure 6:**
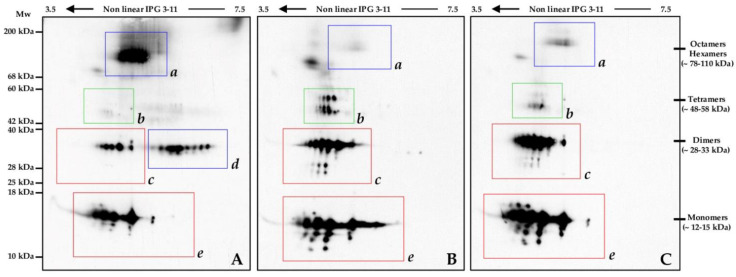
Transthyretin western blot. CSF from pt. 9, before (**A**) and after (**B**) nusinersen therapy, and from Cntl9 (**C**) were resolved by 2D SDS-PAGE and analysed by western blotting using an anti-transthyretin monoclonal antibody. Immunoblot analysis highlighted the general restoring trend of transthyretin isoform pattern to that of the control sample.

The wb images showed a complex pattern of TTR isoforms, with numerous qualitative and quantitative differences occurring between IT0 and IT1/Ctrl 9 samples. Detected signals clustered into five main areas, highlighted in Figure 6 by rectangles and italic letters (*a-e*). In general, a consistent similarity was observed between the IT1 and Ctrl 9 samples, with the exception of the isoelectric series in the *b* area at Mw between 42 and 60 kDa, Figure 6B,C-green rectangle. These proteoforms presented higher signal intensities in IT1 than in Ctrl 9.

Signals much more intense or even present only in IT0 localized in two areas at medium-high and high Mw, which were highlighted by blue rectangles: *a* (68–200 kDa) and *d* (28–40 kDa) areas in Figure 6.

The *b*, *c* (25–40 kDa), and *e* (10–18 kDa) areas showed numerous qualitative and quantitative differences occurring between IT1/Ctrl 9 and IT0, with a consistent reduction of immunoreactive signals in IT0.

The principal immunoreactive isoelectric series we detected had Mw corresponding to TTR monomers (e area), dimers (c and d areas), tetramers (b area), and other oligomers (a area), maybe hexamers and octamers (Figure 6). It is interesting to note that in IT1 and Ctrl 9 the high decrease of the a area signals corresponded to a qualitative and quantitative increase of immunoreactive spots in the b, c, and e areas. Moreover, TTR proteoforms in the d area suggested the occurrence in IT0 of differential co- and post-translational modifications, not observed in IT1 and Ctrl 9. According to literature [32,33], we may indeed speculate that in pt. 9 CNS aberrances triggered by the pathology could have affected the biochemical properties of TTR, during its synthesis or in the extracellular environment, thus to alter its function and stability. A resultant dissociation of native tetrameric TTR and consequent release of monomers may have caused non-physiological oligomerization of these latter, as described in TTR amyloidoses [33]. Oligomers or their aggregates may be more difficult to solubilize than the native tetrameric TTR and they may have led, at least in part, to the isoforms spanning from 78 to 110 kDa that we detected in the *a* area of the IT0 sample (Figure 6). In pt. 9, the nusinersen induced CNS improvements evidently revert the TTR 2D pattern and, bona fide, its properties and functions to those of the control subject n. 9.

### 2.6. Oxidized Protein Pattern in Two SMA Type 1 Patients before and after Nusinersen Therapy

The oxidative stress was found involved in some aspects of SMA neurodegeneration [34], primarily due to the decrease in mitochondrial membrane potential [35] and to the worsening of the inflammatory condition, which result from the SMN depletion [7].

Protein oxidation is a hallmark of oxidative stress. The majority of protein oxidative modifications is irreversible and seriously affects protein structure and functions, thus often leading to insoluble protein aggregates [36]. ROS attack to specific amino acid side chains causes the formation of carbonyl groups (aldehydes and ketones). These are generated early during oxidative stress and are quite stable. Consequently, carbonyl groups may be used to evaluate the oxidative stress injury [37].

Hence, to investigate whether oxidative stress is affected by the therapy, we visualized the protein carbonyl-group profiles, as indicative of protein oxidation, in CSF samples from pts 9 and 10 at baseline (IT0 and JT0) and after six months of nusinersen treatment (IT1 and JT1). We also included in the experiment CSF samples from Ctrls 3 and 10 to assess differences in carbonyl-group occurrence between two SMA type 1 patients and two non-SMA controls.

**Figure 7:**
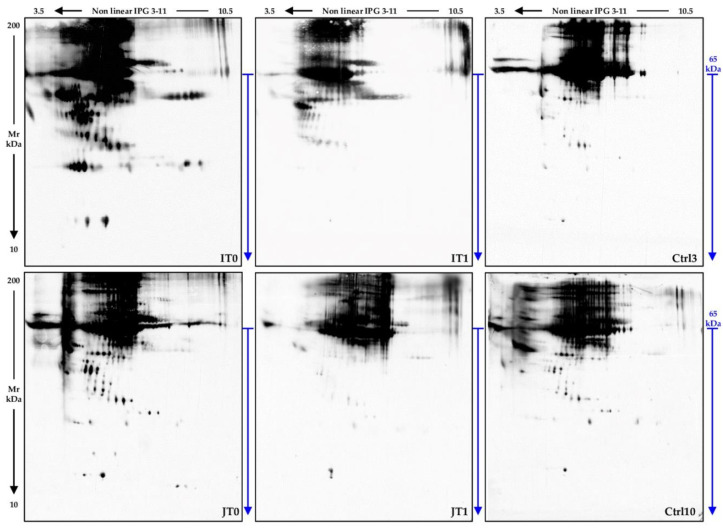
Oxidized protein profile of CSF samples from patients 9 and 10, before and after nusinersen therapy, and from two control subjects. Western blot analysis with an anti-DNP antibody on CSF samples from pts 9 and 10, before (IT0 and JT0) and after (IT1 and JT1) nusinersen therapy, and from Ctrls 3 and 10 (Ctrl 3 and Ctrl 10). 2DE immunoblot analysis showed an evident reduction of carbonylated protein species after six months of nusinersen treatment. This signal decrease is mainly evident for protein species having a low-medium Mw (gel area marked by the blue arrows).

Figure 7 shows the general decreasing trend in signal intensity of oxidized proteins in IT1 and JT1 when compared with IT0 and JT0, respectively. In particular, several of the proteoforms resolved in IT0 and JT0 at low-medium Mw values (Figure 7, below the blue bar), which were also present in Ctrls, were not detected in T1 samples.

These results suggested that oxidative stress in CSF from pts 9 and 10 reduced after nusinersen treatment, thus making us suppose an improvement in inflammatory response, as previously proposed by Ando et al. [7].

## 3. Discussion

The spontaneous course of SMA type 1 does not expect any clinical improvement over time, as this is a progressive disease characterized by a rapid degeneration of motor neurons. The recent advent of the ASO nusinersen has significantly improved life expectancy and the motor abilities of SMA type 1 infants by ameliorating motor functions and bringing assisted ventilation independence [19,21].

SMA type 1 patients enrolled in our study showed a significant improvement in motor functions after a relatively short period of the nusinersen therapy. Previous studies, focused on identifying potential biomarkers of disease progression and treatment response in SMA type 1 patients, described a decrease of the axonal-damage marker neurofilaments after ASO therapy, both in cerebrospinal fluid (CSF) and in blood [23,38]. This evidence was not confirmed in the later-onset SMA where the slower disease progression might impede the detection of biomarker changes [39].

To date, the only reported CSF proteomic study in nusinersen treated SMA patients was performed in adult SMA 2 and 3 probands [24] and no protein was differentially detected in response to 10 months of treatment.

Conversely, in our pediatric cohort, we found a generalized variance decrease among CSF proteomic patterns from treated patients and controls and a total of 28 protein spot differences among SMA type 1 CSF before (T0) and after 180-day nusinersen treatment (T1) (Figure 1 and Table 2). As evidenced by principal component analysis and hierarchical clustering (Figure 2 and Figure 3, respectively), nusinersen actually induced changes in CSF protein profiles, with a restoring trend for the 42% of the detected protein differences (Supplementary Table S1).

The 17 spot differences we identified by MS corresponded to different proteoforms of 6 unique proteins, as detailed in Table 2. Since differential co- and posttranslational modifications modify physical and biochemical properties of proteins and indeed their localization and function, six months of nusinersen treatment evidently modulate, in our preliminary investigation, the biomolecular and functional properties of the CSF by inducing variations in both the quality and quantity of liquor protein isoforms. As CSF composition reflects central nervous system (CNS) physiology, identified differences evidenced a nusinersen induced neuronal response that we attempted to understand by investigating BP GO-terms, which annotate identified differences. The REVIGO tool was hence applied to summarize and semantically cluster GO-terms (Figure 4 and Supplementary Figure S1), and ASO-affected CSF-proteins resulted regulating numerous cellular functions, despite their extracellular localization. This indeed supported the hypothesis to assess functional defects of neuronal cells, and their eventual nusinersen induced restoring, by investigating CSF protein profiles from SMA type 1 probands. In particular, the obtained plot highlighted identified proteins exerting vital functions for neuronal physiology in the central and even peripheral nervous system.

### 3.1. Are APOE and APOA1 Crucial Allies of Nusinersen in Restoring Nervous System Plasticity in SMA Type 1 Patients?

APOA1 and APOE are the two identified protein differences having the neurological implications highlighted by the REVIGO analysis. They are multifunctional proteins mainly known for regulating lipid homeostasis in plasma and tissues. Since they represent the major lipoproteins in CSF, APOE and APOA1 are fundamental for cholesterol and other lipid trafficking in CNS and are indeed considered relevant for physiological synaptogenesis and neurite growth as well as for their maintenance [40,41].

Lipids comprise more than the 50% of the brain dry weight and provide, beside energy, essential structural and functional roles in the nervous system. They actually compose cellular membranes and modulate several neuronal properties by supporting their integrity, isolation/permeability, plasticity, and lipid raft organization for growth factor signal transduction, cell adhesion, axon guidance, and synaptic transmission [42]. Lipids may be also rapidly metabolized to neuroactive messengers and to mediators of inflammation [43]. It is therefore not surprising that several neuronal and systemic processes depend on lipid metabolism and homeostasis and that lipid dysfunctions, observed also in SMA [44], are associated with neurodegeneration [40,45,46,47]. Accordingly, defects in APOE and APOA1 regulation may be involved in the pathophysiological processes at the bases of various nervous disorders.

Although our study enrolled a limited number of cases and further analyses are needed to confirm the use of the identified proteins as biomarkers, the differences of APOE and APOA1 isoforms that we detected occurring among T0 and control samples may be associated with nervous system defects caused by the disease. Consequently, the general restoring trend of APOA1 isoform abundance to the corresponding control values and the overall protein abundance increase of APOE are bona fide considered a nusinersen direct or indirect effect that may contribute to improving the patient outcome.

Outside their trophic role in lipid trafficking and metabolism, APOE and APOA1 are in fact involved in numerous cellular processes, many of which have been reported as aberrant in SMA and other degenerative disorders of the CNS.

APOE, which is produced beside astrocytes and microglia also by stressed or damaged neurons, is engaged in lipid re-cycling and re-distribution for damage repair and inflammation modulation [48,49,50]. In addition, neurological impairments related to its ε4 variant prove [49,51,52,53] that APOE is fundamental for development, sprouting, migration, resilience, and survival of neurons [48,52].

CSF APOA1 derives from plasma filtering through the blood-brain barrier (BBB) and, while CNS APOE vehicles locally produced lipids, it is supposed to transport “systemic” lipids from plasma to the CSF [54,55,56,57], thus acting as a messenger from blood to CNS [58,59].

APOE and APOA1 induce several neuron responses by binding to different cellular membrane receptors [60,61]. This aspect is consistently stressed by the *REVIGO signal transduction* cluster (iv) where practically all the included GO-terms annotate APOE and APOA1. These latter trigger in fact a number of intracellular signaling cascades by modulating various kinases [62,63], which are active in CNS physiological and pathological states. Among them, the extracellular signal-regulated kinase 2 (ERK2) [GO term: positive regulation of ERK1 and ERK2 cascade, cluster (i)] promotes, in physiological conditions, neurogenesis and neuronal differentiation while the extracellular signal-regulated kinase 1 (ERK1) protects neurons from N-methyl-D-aspartate (NMDA) induced death [GO term: NMDA glutamate receptor clustering, cluster (ii)] and regulate autoimmune reactions [64]. Interestingly, APOE was described regulating the c-JUN N-terminal kinase (JNK), ERK, and p38 (JNK/ERK/p38) pathway in regenerated axon after traumatic brain damage [65]. In addition, the APOA1 increase observed during the secondary phase of traumatic spinal cord injury [66] was supposed promoting healing processes and axonal repair in the CNS by inducing ERK pathway and actin polymerization [61]. This role of APOA1 in neuro-regeneration and repair [67] was strengthened by its decreased concentrations occurring in serum and CSF of neuro-degenerative disorders [57,68,69]. Therefore, nusinersen related APOE and APOA1 modulation may contribute towards reducing SMA impairment by improving neuronal plasticity and regeneration and by reducing immune responses.

As further confirmation of this, APOE has been described inducing the synthesis of cyclic guanosine-3′,5′-monophosphate (cGMP) and nitric oxide (NO) [GO-term: cGMP-mediated signaling, cluster (iv)] [70,71] thus mimicking neurotrophic factors for neuron protection and survival [72].

Moreover, APOE and APOA1 are involved in the regulation of the Rho family of small G-proteins [GO terms: G protein-coupled receptor signaling pathway; positive regulation of Rho protein signal transduction, cluster (iv)], including Cdc42 [GO term: regulation of Cdc42 protein signal transduction, cluster (iv)], Rac1, and RhoA [61,63]. Given the control exerted by these small GTPases on cytoskeleton [GO: cytoskeleton organization, cluster (vi)] for stress fibers formation [GO term: positive regulation of stress fiber assembly, cluster (vi)], axonal growth, cone dynamics and neurite branching, APOA1 and APOE may contribute in regulating and maintaining morphology and polarity of neurons and, indeed, in neuronal differentiation, integrity and functionality [73,74]. In particular, APOE is known reducing RhoA activation [75] and APOA1 has been reported modulating actin polymerization by activating Cdc42 [61]. These processes are crucial in the formation and maintenance of motor neurons and of the neuromuscular junctions (NMJ), as observed in a mouse model of SMA where increased activation of RhoA/ROCK pathway have been described concurring to neuritogenesis defects, or in *Smn1*-knockdown PC12 cells where RhoA and Cdc42 dysregulation have been reported [76,77].

The SMN protein localizes in neurites and in growth cones, and its absence/depletion in axons of motor neurons causes defects in NMJ formation and maturation, with consequent degeneration of the neuron [78,79]. Reduction of SMN results in a decrease of β-actin-mRNA at growth cones and in the increased activation of RhoA [80]. The consequent actin polymerization imbalance concurs to suppress synapses sprouting and growth at the NMJs [77,81]. Perturbations in generation and functionality of these latter are critical from the early stages of SMA and their aberrances have been described in SMA mice even before the manifestation of the disease [81]. Moreover, pharmacological inhibition of the RhoA/ROCK pathway ameliorates NMJ maturation and improves survival of SMA mice by improving cytoskeleton actin dynamism [80].

Nusinersen can indeed improve survival of neurites and growth cones of motor neurons by restoring cytoskeleton plasticity, for macromolecular and vesicle trafficking and for retrograde and anterograde signals, by acting on Rho GTPase family, directly, via *SMN* expression and, indirectly, via APOE/A1 modulation.

In animal models, SMN deficiency has also been associated to perturbation in ubiquitin homeostasis related to reduced presence and activity of the ubiquitin-like modifier activating enzyme 1 (UBA1) [82]. The deregulation of UBA1, whose mutations have been found in the X-linked infantile SMA [83,84], results in β-catenin accumulation and neuromuscular defects [82]. Accumulation of β-catenin compromises motor neuron functionality deregulating Wnt signaling and, once again, by affecting the actin cytoskeleton organization [85]. In this context, another intriguing function of APOE, which may support nusinsersen therapy, is its capability in inhibiting the canonical Wnt signaling pathway [GO term: negative regulation of canonical Wnt signaling pathway, cluster (iv)] [86,87]. APOE up-regulation may indeed contribute to enhancing cytoskeleton dynamism by supporting SMN also in controlling β-catenin accumulation and, consequently, in improving NMJ regeneration and maintenance.

### 3.2. APOE, APOA1 and Haptoglobin May Support Nusinersen in Inflammation and Oxidative Stress Balancing

Notwithstanding the limited markers and evidences of inflammation in SMA [88], astrogliosis was observed in the spinal cord of SMA patients, thus suggesting inflammatory cytokine release [8]. In addition, Ando et al. have proved SMN protein regulating oxidative stress and inflammatory response in microglia from the SMNΔ7 mouse [7], a SMA animal model where inflammation was previously hypothesized concurring to the progression of the disorder [89]. They also demonstrated that SMN-ASO, having the same structure of nusinersen, blocked microglial activation and reduced oxidative stress in the SMNΔ7 mouse. Glial cell dysfunctions have been recently proposed promoting the pathogenesis of SMA: a reciprocal stimulation between activated astrocytes and microglia has been actually hypothesized inducing neuroinflammation. This results in reactive oxygen species (ROS) and pro-inflammatory cytokine production and release, immune infiltration and pro-apoptotic pathway activation [90]. Notably, SMA motor neurons have been suggested more vulnerable to TNFα induced death [7]. Therefore, an increased cytokine release may be detrimental for SMA probands.

The generalized APOE up-regulation we detected and the recovery trend of APOA1 isoform pattern may support, in a positive feedback, the inflammation and oxidative stress reduction induced by nusinersen. APOE has been in fact described to down-regulate astrocyte activation and to modulate microglia activation in vitro. It indeed controls inflammatory gene expression in CNS (e.g., Il-1β, IL-6, and TNFα) and neurotoxicity that arises from the glial innate immune response [GO term: regulation of innate immune response, cluster v)] [50,91]. In addition, APOE may act a key role in protecting spinal motor neurons against ROS damage by up-regulating cGMP [92].

Interestingly, APOE also exerts an anti-inflammatory effect on cerebral microvasculature by influencing pericyte cerebrovascular-control on blood flow [GO term: vasodilation, cluster vii)] and on the integrity of the blood–brain barrier (BBB), whose defective permeability is still debate in SMA [75,93]. APOE may indeed reduce the risk of immune infiltration in damaged CNS.

Similarly, APOA1 has been described exerting an anti-inflammatory role [GO term: negative regulation of inflammatory response, cluster (v)] when associated with high-density lipoprotein (HDL). By inhibiting Il-1β and TNFα exocytosis [GO: negative regulation of cytokine production involved in immune response, cluster (iv], plasmatic APOA1 interferes with macrophage and T cell communication, thus preventing T cell activation [94,95]. This lipoprotein also inhibits cytokine release and oxidative burst in neutrophils [96], hence down-regulating inflammation and ROS injury [58].

Besides APOE and APOA1, nusinersen treatment also induced abundance changes of haptoglobin (HP) and variations in haemoglobin (Hb) proteoform pattern (Figure 1 and Table 2). HP is a type 2 acute phase response protein synthesized by the liver [97] and that is normally up-regulated in inflammatory conditions [98]. It protects CNS from inflammation and neurodegeneration. In fact, its high affinity binding with free neurotoxic Hb reduces heme-induced oxidative stress [99] and promotes Hb removal via CD163 mediated endocytosis in neurons, astrocytes, and microglial/macrophages [100,101]. In addition, haptoglobin inhibits prostaglandin pathway and endothelium relaxation, by limiting Hb interactions with prostaglandin synthase and NO [102,103]. It protects APOA and APOE from oxidative alteration [104], hence preserving APOA1 anti-inflammatory potential [58].

Noteworthy, increased HP-levels were found in CSF of patients affected by several neurological diseases, such as traumatic brain injury [105], AD [106], multiple sclerosis [107], Guillain-Barré syndrome [108], and Huntington’s disease [109].

Therefore, we may hypothesise that the haptoglobin increase in T0 samples may result from a neuroprotective mechanism against Hb injury in central nervous system [110]. Conversely, the HP abundance decrease at T1 may suggest its consumption during Hb clearance or may indicate an inflammation decrease induced by nusinersen.

Markers of oxidative injury detected in SMA patients have been related to inflammatory and neurodegenerative events [88]. Activated glial cells and astrocytes produce toxic molecules, such as ROS and NO, which may induce apoptosis in nearby neurons [111,112]. In fact, ROS/reactive nitrogen species (RNS) overpresence causes oxidative stress that enhances inflammatory reaction and, on the other hand, lipid peroxidation, DNA damage, and protein modifications [113]. Lipid peroxidation occurs on polyunsaturated fatty acids, which are highly abundant in CNS and in mitochondrion membranes [114]. Miller et al. [35] reported high oxidation levels in SMNΔ7 mice motor neurons related with reduced mitochondrial membrane potential. This could trigger cytochrome c release, generation of free radicals and onset of apoptosis [115], with detrimental effects on CNS.

According to the REVIGO analysis, identified protein differences resulted positively involved in redox balancing [GO term: cellular oxidant detossification, cluster (iii); and GO terms: response to reactive oxygen species; response to oxidative stress, cluster (v)] and their nusinersen related modulation may be referred as an indirect effect of the ASO on oxidative stress/inflammation reduction.

The therapy evidently induced changes in the CNS of the SMA type 1 patients we investigated that in pts 9 and 10 probably resulted in inflammation and oxidative stress reduction, as suggested by the decrease of their CSF protein carbonylation (Figure 7). Despite preliminary, this result is indicative of a general trend of oxidative reduction apparently associated to the therapy.

### 3.3. Nusinersen Treatment Highly Influences Transthyretin Pattern in the Investigated Cohort of SMA Type 1 Patients

TTR is a serum and CSF protein with metalloprotease activity [116]. It is one of the most abundant proteins in CSF [117] and, despite it is best known for transporting thyroxine (T4) and retinol through the blood–brain barrier (BBB) [118], TTR has been suggested operating a broad range of functions in the nervous system physiology.

Investigations in murine models evidenced a TTR critical activity in peripheral nerve functions and regeneration and a neuroprotective role in the CNS. Here, TTR seems improving behavior and cognitive performance and inducing neurite outgrowth [119]. In patients with stroke, TTR has been described as a positive prognostic indicator of clinical outcome [119]. Confirming this, dysregulations of TTR levels characterize several neurological disorders [120,121,122,123,124,125]. In particular, the mutant and wild type TTR amyloidoses are human disorders caused by TTR misfolding, native tetrameric structure lost, and insoluble aggregate formation and deposition in different organs [33].

Motor neurons are also known secreting TTR [126] and its absence was demonstrated causing sensorimotor deficits and nerve regeneration reduction [127,128]. The higher levels of TTR found in ALS patients have been supposed a regenerative response attempt against the neuronal injury caused by the pathology [120].

The TTR up-regulation in the nuisinersen-treated SMA type 1 patients we investigated may indeed represent a nusinersen-dependent enhancement of the CNS positive reaction to neuronal damage for neuron survival and function restoring. Furthermore, as we evidenced by TTR western blot, the SMA type 1 pt. 9 presented at baseline an aberrant TTR proteoform pattern that broadly reverted to the Ctrl 9 TTR-pattern after six months of nusinersen therapy (Figure 6).

Despite there is no clear evidence proving amyloid deposition in SMA type 1, small oligomers intermediates, which form in early stages of amyloid deposition, are critical for amyloid accumulation in several neurodegenerative disorders. Beyond this, TTR hexamers/octamers may represent, as we hypothesized in the Section 2, for pt. 9 a risk factor for neuron degeneration since small oligomers have been recognized as the major cytotoxic species in TTR amyloidosis [32,33].

In conclusion, in the affected SMA type 1 CNS an eventual TTR up-regulation for coping neurodegeneration may result in oligomer formation. This may reduce TTR neuroprotection potential and, on the other hand, it may increase the cytotoxic risk for the CNS. Improvements described occurring in SMA type 1 after nusinersen administration [17] evidently restore some CNS functionalities that correlate to TTR proteoform pattern reversion to that of non-SMA control and, maybe, to its neuroprotective rules.

TTR proteoform pattern may indeed represent a possible biomarker for monitoring patient responsiveness to the therapy and for predicting SMA type 1 progression. Obviously, further analyzes are needed to confirm what is assumed here on a speculative basis.

### 3.4. Concluding Remarks

Our analyses have evidenced nusinersen modulating CSF protein-pattern in the pediatric SMA-patients we enrolled. As all they were alive, none showed adverse events, but on the contrary, they presented a significant improvement in motor functions, the protein differences we identified occurring between CSF at baseline and after the treatment may be correlated to the disease amelioration. In particular, changes triggered by the ASO to the proteomic profile of apolipoprotein A1, apolipoprotein E, transthyretin, and haptoglobulin contribute to define the molecular bases of the clinical improvements we observed. These proteins are actually known exerting key roles in several of the processes that have been described partially restored by the ASO or that have been reported dysregulated or aberrant in SMA.

Although further analyses are needed to validate in a wider cohort of probands the obtained results, the here identified and discussed proteins may represent precious novel biomarkers for evaluating patient response to nusinersen treatment and disease progression.

## 4. Materials and Methods

### 4.1. Participants and Cerebrospinal Fluid Sampling

The study was approved by the Ethics Committee of the province of Messina. Written informed consent was obtained from all parents of children enrolled in the study.

Ten patients, with a confirmed genetic diagnosis of SMA type 1 underwent the treatment with nusinersen, which was administered through an intrathecal injection by a lumbar puncture, at the recommended dosage of 12 mg/5 mL. Before each nusinersen administration, a cerebrospinal fluid (CSF) volume, equivalent to the ASO dose, was drained, aliquoted, and immediately stored at −80 °C.

Patients received four loading doses at days 0, 14, 28, and 63, and the fifth dose (the first maintaining dose) after further 120 days. Clinical and neurological evaluations of patients were performed at each drug administration by dedicated clinicians. Before the first and the fifth nusinersen injections, CSF samples were obtained. At the same time, a trained physiotherapist assessed patients’ motor function by using the functional Children’s Hospital of Philadelphia Infant Test of Neuromuscular Disorders (CHOP INTEND) scale, which is specific for SMA type 1 patients [129]. Ten children sampled for suspected normal pressure hydrocephalus (NPH), meningitis or Guillain-Barré syndrome were used as controls (age range: 1–36 months). There was no significant difference in age or sex distribution between the SMA and the control groups. Controls had normal CSF cell count, protein, and lactate levels, the suspected diagnosis was not confirmed, and they had no follow-up. Overall, 4 males and 3 females (Ctrls 1–7) were used as controls of proteomic profiles of the first 7 SMA patients (pts 1–7) while further 2 males and 1 female, sampled for meningitis or NPH, were used as controls of patients 8–10.

### 4.2. Cerebrospinal Fluid Preparation for Proteomics Analysis

CSF sample preparation was performed as previously described [123]. Briefly, 333 μL, 666 μL, and 3 mL of CSF were overnight precipitated in 1:4 cold acetone for each analytical and 2D western blot (wb) and Mass Spectrometry (MS)-preparative gels, respectively. After sample/acetone centrifugation at 15,000× *g*, for 10 min at 4 °C, supernatants were removed, and pellets solubilized in 5 μL of 5% (*w*/*v*) SDS and 2.3% (*w*/*v*) DTE. Obtained sample solutions were heated at 95 °C for 7 min by using a test tube dry block heater (GE Healathcare—Billerica, MA, USA). After cooling down to room temperature, a conventional 2D rehydration buffer (8 M urea, 2 M thiourea, 1% (*w*/*v*) DTE, 0.4% (*w*/*v*) CHAPS, with traces of bromophenol blue) was added to each sample to reach a final volume of 350 μL.

### 4.3. Two-Dimensional Gel Electrophoresis

The Two-Dimensional Gel Electrophoresis (2-DE) was performed using the immobiline–polyacrylamide system. IsoElectric Focusing (IEF) was carried out on precast 18 cm long non-linear Immobiline Dry-Strip, with immobilized pH 3–11 gradient (GE Healthcare), and using an Ettan™ IPGphor™ system (GE Healthcare). Immobilized pH gradient (IPG) strips were rehydrated, for 12 h at room temperature with 350 μL of lysis buffer containing bromophenol blue in trace and carrier ampholytes (pH range 3–11), 0.2% (*v*/*v*) for analytical-runs and 2% (*v*/*v*) for MS-preparative runs. Total protein was loaded for the analytical and preparative runs by cup-loading on the Ettan IPGphor Manifold (GE Healthcare). Proteins were focused, at 16 °C, according to the following voltage program: 200 V for 8 h, from 200 V to 3500 V in 2 h, 3500 V for 2 h, from 3500 V to 5000 V in 2 h, 5000 V for 3 h, from 5000 V to 8000 V in 1 h, 8000 V for 3 h, 8000 V up to a total of 90,000 Vh.

After IEF, the IPG gels were equilibrated in 6 M urea, 30% (*v*/*v*) glycerol, 2% (*w*/*v*) SDS, 0.05 M Tris–HCl pH 6.8, 2% (*w*/*v*) DTE for 12 min, and for further 5 min in 6 M urea, 30% (*v*/*v*) glycerol, 2% (*w*/*v*) SDS, 0.05 M Tris–HCl pH 6.8, 2.5% (*w*/*v*) iodoacetamide and trace of bromophenol. The second dimension, SDS-polyacrylamide gel electrophoresis (SDS-PAGE), was performed on 9–16% polyacrylamide linear gradient gels and carried out at 40 mA/gel constant current at 9 °C.

Analytical gels resolving CSF samples at T0 and T1 from pts 1–7 and from Ctrls 1–7 were then stained with ammoniacal silver nitrate, while MS-preparative gels, resolving pooled samples from pts and Ctrls 1–10, were stained according to MS-compatible silver staining protocol as previously reported in [130,131].

All gels were digitalized using the Image Scanner III coupled with the LabScan 6.0 software (GE Healthcare).

### 4.4. Image Analysis and Statistics

SMA samples image analysis was carried out using Image Master 2D Platinum 7.0 software (GE Healthcare). An intra-class analysis was performed matching all gels from the same condition with their reference gel (i.e., the Master gel). In order to find quantitative and qualitative differences, the three Master reference gels were compared in inter-class analysis, assuming as evaluation parameter the relative volume (% Vol), which corresponds to the integration of optical density of a single spot (volume) on the total volume of spots, detected in the same gel, and expressed as a percentage.

Statistical analysis was performed using the Differential Analysis tool in XLStat software (XLSTAT-lifeScience—Paris, France). Then, % Vol differences among matched spots, from the three investigated groups (i.e., Control, T0 and T1), were evaluated by statistical non-parametric Kruskal–Wallis test (*p* ≤ 0.05). The mean rank of significant results was then compared by Dunn’s multiple comparison test. Statistically significant differences were then processed according to the ratio value ≥2 of corresponding % Vols.

Experimental pI and Mw (Da) values were determined by comigration with human serum as internal standard, as previously described [132].

Principal component analysis (PCA) was carried out by XLStat using the overall correlations (% Vol variables) occurring between different spot maps. Samples were plotted into the Cartesian space, by linear transformation, reporting the principal components PC1, PC2, and PC3, which account for the majority of detected variation.

Cluster analysis of the proteomic data was obtained by the HeatMaps function in XLStat using the spot % Vol values and by applying absolute value of linear correlation-based distance (Pearson).

### 4.5. Mass Spectrometry by MALDI-TOF

Differentially abundant spots were manually excised from MS-preparative gels, and destained in 5 mM ammonium bicarbonate and 50% (*v*/*v*) acetonitrile solution and then completely dehydrated in acetonitrile solution. Spots were digested with a 50 mM ammonium bicarbonate and trypsin solution, overnight at 37 °C. Tryptic peptides were applied to a C18ZipTip (Millipore, CA, USA), rinsed with a 0.1% (*v*/*v*) trifluoroacetic acid (TFA) and eluted directly on the MALDI target with 0.5 μL saturated α-cyano-4-hydroxycinnamic acid (1:1 = ACN:0.1% (*v*/*v*) TFA) solution for peptide mass fingerprinting (PMF). Digests were then analyzed with an Autoflex™ Speed mass spectrometer (Bruker Daltonics, Bremen, Germany) equipped with Nd:YAG laser (355 nm; 1000 Hz) operated by FlexControl v3.3 and a 355-nm nitrogen laser. All spectra were obtained by delayed extraction technology in positive reflectron mode and averaged over 100 laser shots to improve the S/N ratio. External high precision calibration was performed using a peptide mixture containing bradykinin (fragment 1–7) 757.39 m/z, angiotensin II 1046.54 m/z, ACTH (fragment 18–39) 2465.19 m/z, Glu fibrinopeptide B 1571.57 m/z and renin substrate tetradecapeptide porcine 1760.02 m/z.

After MS recording, the spectra underwent PMF searching the human SwissProt protein database with Mascot search engine. Applied parameters were: peptide mass fingerprint enzyme: trypsin; fixed modification: carbamidomethylation (Cys); variable modifications: oxidation of methionine; mass values: monoisotopic. The ion charge state was set at +1; maximum missed cleavages were set at 1; mass tolerance was set at 100 ppm.

### 4.6. Gene Ontology (GO) Clustering

Biological Process (BP) Gene Ontology (GO) terms, annotating the differentially abundant proteins identified among T0 and T1 and control groups, were retrieved from UniProtKB (http://www.uniprot.org/; 2020_06 release: 209,721,111 UniProt/TrEMBL entries) and by applying QuickGO browser [133,134] (http://www.ebi.ac.uk/QuickGO/). As previously described [135], redundancy reduction of the GO-term list was performed considering semantic similarity and by applying the clustering/summarizing algorithm of the REVIGO resource (http://revigo.irb.hr/; accessed on December 2020) [136]. A medium large GO list was obtained by setting the cut off value at 0.7. Homo sapiens was selected as reference database with GO term sizes. Finally, the resulting non-redundant BP GO-term set was visualized through the scatterplot visualization option supported by the REVIGO server.

### 4.7. Western Blot

For APOE monodimensional (1D) wb, 333 μL of CSF samples from patients 8, 9, and 10 (Table 1) at baseline (HT0, IT0, and JT0, respectively) and after six months of nusinersen therapy (HT1, IT1, and JT1, respectively), and from 3 control subjects (Ctrl 3, 5, and 9) were overnight precipitated in 1:4 cold acetone. After sample/acetone centrifugation at 15,000× *g*, for 10 min at 4 °C, sample pellets were resuspended and denatured in Laemmli buffer: 100 mM Tris–HCl pH 6.8, 2% (*w*/*v*) SDS, 20% (*v*/*v*) glycerol, 4% (*v*/*v*) β-mercaptoethanol, and heated at 95 °C for 5 min [137]. The same procedure was applied for CSF aliquots (ranging from 222 to 150 μL) from patients and controls previously investigated by 2D-PAGE/MS: pts 1, 3, 4, 5, 6, 7, and Ctrl 1, 2, 3, 5, 6. These samples were used for APOE and APOA1 1D wb.

Twenty micrograms from each sample were then separate by 1D SDS-PAGE and subsequently transferred onto a nitrocellulose membrane (GE Healthcare), as previously described [138].

Meanwhile, HT0-HT1 and Ctrl 8, and IT0-IT1 and Ctrl 9 samples were applied for 2D wbs of APOA1 and transthyretin, respectively. IT0-IT1, JT0-JT1, and Ctrls 3 and 10 samples were analysed for the staining of carbonyl-groups. Wb preparative gels, obtained as above described in *4.3 Two-Dimensional Gel Electrophoresis*, underwent wet blotting according to Towbin [138].

Reversible Ponceau S staining [0.2% (*w*/*v*) Ponceau S in 3% (*v*/*v*) trichloroacetic acid (TCA)] was used in order to check the gel resolution quality, and protein loading and transfer. In 2D wb, Ponceau S membrane staining was also applied for detecting landmark spots to properly match digitalized images of chemioluminescence exposed photographic films with silver stained 2D gels. This allowed defining silver stained spots, corresponding to immunoreactive proteins in CSF electropherograms [139]. As wb is more sensitive than silver staining, signal correspondence between gel-spots and photographic film-signals is possible merely when protein abundance of gel-spots exceeds the detection threshold of silver staining.

Nitrocellulose membranes were blocked in 3% (*p*/v) dried skimmed milk, 0.1% (*v*/*v*) triton X100 in PBS pH 7,4 and then overnight incubated, at 4 °C, with primary antibodies. A 1:1000 dilution of a mouse monoclonal anti-APOE antibody (Ab) (sc53570), from Santa Cruz Biotechnology (San Jose, CA, USA), was applied to evaluate the oval abundance of APOE in the 17 samples [*n*(T0) = 6, *n*(T1) = 6, and *n*(Ctrl) = 5] analyzed by 1D wb. HT0/T1, and Ctrl 8 2D membranes and ET0/T1, FT0/T1, GT0/T1, and Ctrl 1–2 1D membranes were incubated with a mouse anti-APOA1 monoclonal-antibody (Ab) (sc376811) (Santa Cruz Biotechnology), diluted 1:1000. IT0, IT1, and Ctrl 9 membranes were probed with a mouse monoclonal anti-transthyretin Ab (sc377178) (Santa Cruz), diluted 1:1000. Successively, membranes were thoroughly washed in the milk blocking solution and incubated, for further 2h at room temperature, with a goat peroxidase-conjugated polyclonal anti-mouse IgG Ab (172–1011) (Bio-Rad, Hercules, CA, USA), diluted 1:3000 and applied as secondary antibody.

Since image analysis did not detect changes of albumin abundance in 2D silver stained gels, we performed albumin immunoblotting for ensuring equal sample loading in each lane of the 1D membrane. A rabbit monoclonal anti-albumin Ab (126584) from Calbiochem (San Diego, CA, USA), 1:2000 diluted, was used as primary antibody. A goat peroxidase-conjugated polyclonal anti-rabbit IgG (A4914) (Sigma-Aldrich—Saint Louis, MO, USA), diluted 1:10000, was used as secondary Ab.

For reactive carbonyl derivatives detection, isoelectrofocused proteins, from pts 9–10 (T0/T1) and Ctrls 3/10, were derivatized by using the carbonyl reagent 2,4-dinitrophenylhydrazine (DNPH). After IEF, strips were rinsed with water and incubated in a solution containing 5% (*v*/*v*) TCA and 10 mM DNPH, for 20 min at room temperature. Then, derivatizated strips underwent two steps, of 5 min each, of neutralization by incubation in a washing solution containing 8 M urea, 20% (*v*/*v*) glycerol, 1% (*w*/*v*) SDS and 150 mM Tris-HCl pH 6.8. After that, strips were equilibrated as described in *4.3 Two-Dimensional Gel Electrophoresis* and the second dimension was performed. The immunodetection of derivatizated carbonyl-groups was obtained by using a rabbit polyclonal Ab anti-2,4-dinitrophenyl (DNP) (D 9656) (Sigma), dilution 1:10,000, and an anti-rabbit IgG (Sigma), diluted 1:10,000 and used as secondary Ab.

Immunoreactive bands and spots were visualized by chemiluminescence using ECL detection reagents (GE Healthcare). Chemiluminescent signals were captured by ChemiDocTM imaging system (Bio-Rad) and/or detected by exposing membranes to Hiyperfilm ECL X-ray films (GE Healthcare). Wb images were analyzed by using the ImageQuant v. 3.0 software (Molecular Dynamics World Headquarters—Sunnyvale, CA, USA) and the ImageMaster 2D Platinum v. 6.0 software.

Statistic differences of normalized mean relative-integrated-density values (computed for individual immunoreactive bands) between T0, T1, and Ctrl groups were evaluated, for the 1D wb, by one-way ANOVA. A pairwise comparison of experimental groups was also performed by the Tukey’s post-hoc multiple comparison test using the Exel Template inerSTAT-a 2.0.

## Figures and Tables

**Table 1:** Clinical data of SMA type 1 patients (pts) 1–10, including score of the CHOP INTEND (Children’s Hospital of Philadelphia Infant Test of Neuromuscular Disorders) scale at baseline (T0) and at 180-days (T1) after nusinersen treatment initiation.

Pts ^a^	Sex M/F	Age at Symptoms Onset (Months)	Age at Treatment Onset (Months)	CHOP INTEND T0	CHOP INTEND T1	ΔCHOP INTEND (T1-T0)	*SMN2* Copy Number	SMA Type 1 Decimal System ^b^	SMA Type 1 ABC System ^c^	Respiratory Support ^d^ T0 (Hours)	Nutritional SUPPORT ^e^ T0	Respiratory Support ^d^ T1 (Hours)	Nutritional Support ^e^ T1	Sample T0	Sample T1
**Pt. 1**	M	0	2	21	35	14	2	1.5	A	NIV (12)	None	NIV (18)	NGT	AT0	AT1
**Pt. 2**	M	3	17	22	35	13	3	1.5	B	Nocturnal NIV (8)	PEG	Nocturnal NIV (8)	PEG	BT0	BT1
**Pt. 3**	M	5	25	43	46	3	3	1.9	C	Nocturnal NIV (8)	None	Nocturnal NIV (8)	None	CT0	CT1
**Pt. 4**	M	5	28	42	47	5	3	1.9	C	None	None	None	None	DT0	DT1
**Pt. 5**	F	4	7	39	48	9	2	1.9	C	Nocturnal NIV (8)	None	Nocturnal NIV (8)	None	ET0	ET1
**Pt. 6**	F	3	5	24	35	11	2	1.5	B	Nocturnal NIV (8)	PEG	Nocturnal NIV (8)	PEG	FT0	FT1
**Pt. 7**	M	2	3	30	48	18	2	1.5	B	Nocturnal NIV (6)	None	Nocturnal NIV (4)	None	GT0	GT1
**Pt. 8**	M	2	3	23	36	13	2	1.5	B	Nocturnal NIV (8)	PEG	Nocturnal NIV (8)	PEG	HT0	HT1
**Pt. 9**	M	0	2	16	36	10	2	1.1	A	NIV (24)	PEG	NIV (24)	PEG	IT0	IT1
**Pt. 10**	M	1	3	9	35	16	2	1.5	A	NIV (10)	OGT	NIV (18)	PEG	JT0	JT1

**^a^** Patients (pts). **^b^** Decimal system is a SMA type 1 classification, according to decreasing clinical severity (1.1, 1.5, 1.9) [29,30]. **^c^** ABC system is a SMA type 1 classification based on age of symptom onset and according to decreasing clinical severity (A, B, C) [31]. **^d^** All patients requiring respiratory support received a non-invasive ventilation (NIV). **^e^** All patients needing an enteral nutritional support had a percutaneous endoscopic gastrostomy (PEG) performed, with the exception of two patients (pt.1 and pt.10), who had a nasogastric tube (NGT) or an orogastric tube (OGT) feeding, respectively at T1 and T0.

**Table 2:** Significant protein-spot differences, from T0 vs. T1, Control vs. T0, and Control vs. T1 comparisons, identified by mass spectrometry.

Spot N. ^a^	Protein Description ^b^	Ctrl Spot Mean % Vol ± SD ^c^	T0 Spot Mean % Vol ± SD ^c^	T1 Spot Mean % Vol ± SD ^c^	AC ^d^	Experimental pI; Mw ^e^	Theoretical pI; Mw ^f^	Mascot Search Results ^g^		
								Score	No. of Matched Peptides	Sequence Coverage (%)
31	Apolipoprotein A1	174 ± 46 #	73 ± 19 #	102 ± 38	P02647	5.22; 24559	5.56; 30777	181	24	61
33	Apolipoprotein A1	238 ± 63 #	114 ± 48 # §	234 ± 43 §	P02647	5.34; 24211	5.56; 30777	170	21	56
17	Apolipoprotein E	120 ± 49 # *	257 ± 106 #	254 ± 148 *	P02649	5.49; 36422	5.65; 36154	111	18	45
19	Apolipoprotein E	506 ± 191 # *	214 ± 149 #	242 ± 128 *	P02649	5.52; 34722	5.65; 36154	82	13	29
34	Hemoglobin sub. β	376 ± 163	566 ± 42 §	213 ± 107 § #	P68871	6.31; 24629	6.74; 15998	99	12	82
35	Hemoglobin sub. β	462 ± 294 *	0	210 ± 179 *	P68871	6.46; 24629	6.74; 15998	56	6	39
36	Hemoglobin sub. β	72 ± 50	0	139 ± 88	P68871	7.65; 24073	6.74; 15998	83	8	62
49	Hemoglobin sub. β	123 ± 141 #	1284 ± 1151 #	928 ± 983	P68871	6.75; 12024	6.74; 15998	100	10	80
50	Mix: Hemoglobin sub. β Hemoglobin sub. α	57 ± 30	0	0	P68871 P69905	7.55; 12589	6.74; 15998 8.72; 15257	119 58	12 6	82 42
51	Hemoglobin sub. α	91 ± 79 # *	522 ± 128 #	485 ± 260 *	P69905	7.95; 12589	8.72; 15257	100	9	50
53	Hemoglobin sub. α	46 ± 52 # *	294 ± 103 #	565 ± 303 *	P69905	8.42; 12686	8.72; 15257	104	9	50
54	Hemoglobin sub. α	93 ± 31 *	172 ± 111	254 ± 214 *	P69906	7.55; 12116	8.72; 15257	70	7	50
16	Haptoglobin	152 ± 96	185 ± 102 §	44 ± 37 §	P00738	5.49; 41071	6.13; 45205	54	11	25
20	Transthyretin	278 ± 331 #	15 ± 14 # §	116 ± 82 §	P02766	5.39; 30717	5.49; 15887	67	8	65
43	Transthyretin	533 ± 203 # *	129 ± 60 # §	263 ± 137 * §	P02766	5.17; 14121	5.49; 15887	70	8	50
46	Transthyretin	145 ± 49 *	227 ± 87	315 ± 143 *	P02766	5.54; 12816	5.49; 15887	116	10	65
48	Transthyretin	19 ± 12 *	27 ± 16	54 ± 37 *	P02766	5.52; 11902	5.49; 15887	95	10	65

**^a^** Spot numbers match those used in Figure 1 to indicate protein-spot differences; **^b^** UniProt protein name; **^c^** Mean % Vol and standard deviation (SD) values computed for identified spot-differences matched in intra-class analysis; **^d^** UniProt accession number; **^e^** Experimentally determined pI and Mw using human serum as internal standard; **^f^** Predicted pI and MW according to protein sequence as computed by the Compute pI/Mw tool (http://web.expasy.org/compute_pi/); **^g^** Mascot search results: number of matched peptides, sequence coverage, and score. Protein differences were considered significant when they showed both statistical reliability and, at least, 2-fold change in expression: **#** Ctrl vs. T0; * Ctrl vs. T1; **§** T0 vs. T1.

## Supplementary Materials

Supplementary materials can be found at https://www.mdpi.com/article/10.3390/ijms22094329/s1. Figure S1: REVIGO scatterplot of Biological Process GO-terms annotating the identified differences, with neuronal BP terms reported near the corresponding cluster. Figure S2: APOE 1D western blot on CSF samples from pts not investigated by 2D-PAGE/MS (pts 8–10) and Ctrl subjects investigated and not investigated by 2D-PAGE/MS (Ctrl 3, 5 and 9, respectively), and corresponding statistical analysis. Table S1: Significant protein-spot differences detected in Ctrl vs. T0, Ctrl vs. T1 and T1 vs. T0 comparisons. Table S2: REVIGO summarized BP GO-terms.
