# Supplementary material for: Nusinersen Modulates Proteomics Profiles of Cerebrospinal Fluid in Spinal Muscular Atrophy Type 1 Patients
Source: Int J Mol Sci. 2021 Apr 21;22(9):4329. doi: 10.3390/ijms22094329 (PMC8122268; doi:10.3390/ijms22094329)
Supplement: Supplementary file 1 [file ijms-22-04329-s001.zip › Supplementary Table S1.pdf]

Supplementary Table S1: Significant protein-spot differences detected in Ctrl vs T0, Ctrl vs T1 and T1 vs T0 comparisons according to statistics (Kruskal Wallis test = p≤0.05) and sample ratio ≥ 2.

Spot number	Ctrl spot mean %Vol ± SD	T0 spot mean %Vol ± SD	T1 spot mean %Vol ± SD
1	194±129*	210±100§	21±23 *§
2	97±68	122±53§	59±35§
3	97±61	0	38±18
4	166±57#*	52±57#	39±10*
5	104±105	0	0
6	50±30	117±91§	53±23§
7	301±209	0	0
8	173±72*	125±61	71±35*
9	567±241#*	147±114#	199±138*
10	105±88	0	72±44
11	143±27#*	66±31#	58±40*
12	42±34	0	0
13	89±85	163±115	0
14	131±96	185±102§	45±30§
15	0	71±30	112±102
16	152±96	185±102§	44±37§
17	120±49#*	257±106#	254±148*
18	61±26	73±37§	25±17§
19	506±191#*	214±149#	242±128*
20	278±331#	15±14#§	116±82§
21	230±187	69±25§	140±57§
22	104±134	0	17±12
23	40±38	0	67±93
24	38±22	0	12±8
25	86±36*	52±30	40±17*
26	37±26	34±26	0
27	69±51	0	0
28	67±35	59±14	0
29	103±35	32±20§	97±58§
30	179±99	0	65±66
31	174±46#	73±19#	102±38
32	136±131	0	0
33	238±63#	114±48#§	234±43§
34	376±163	566±42§	213±107§#
35	462±294*	0	210±179*

36	72±50	0	139±88
37	10±13	0	7±8
38	9±7	0	10±8
39	9±9	0	9±7
40	57±37	0	0
41	26±10#	64±25#	48±21
42	199±116#*	35±20#	83±55*
43	533±203#*	129±60#§	263±137*§
44	42±25	0	0
45	92±27	0	0
46	145±49*	227±87	315±143*
47	39±25	51±31§	101±42§
48	19±12*	27±16	54±37*
49	123±141#	1284±1151#	928±983
50	57±30	0	0
51	91±79#*	522±128#	485±260*
52	39±37#*	270±96#	316±351*
53	46±52#*	294±103#	565±303*
54	93±31*	172±111	254±214*
55	40±23	0	18±12
56	48±23	0	20±11
57	34±23	0	39±19

Protein differences were considered significant when they showed both statistical reliability and, at least, 2 fold change in expression: # Control (Ctrl) vs T0; * Ctrl vs T1; § T0 vs T1.
