# Supplementary material for: Nusinersen Modulates Proteomics Profiles of Cerebrospinal Fluid in Spinal Muscular Atrophy Type 1 Patients
Source: Int J Mol Sci. 2021 Apr 21;22(9):4329. doi: 10.3390/ijms22094329 (PMC8122268; doi:10.3390/ijms22094329)
Supplement: Supplementary file 1 [file ijms-22-04329-s001.zip › Supplementary Table S2.pdf]

Supplementary Table S2: REVIGO summarized BP GO-terms visualized by bubbles in the REVIGO scatterplot (Fig. 4 in the main text and Supplementary Fig. S1 in Supplementary Material). GO terms are listed grouped in the 7 clusters that were delineated according to term semantical similarity.

GO terms with neurological implications #	Biological Processes GO terms	GO identifier
	**Metabolism and gene expression**	
1	**Amyloid precursor protein metabolic process**	**GO:0042982**
	Endothelial cell proliferation	GO:0001935
	ERK1 and ERK2 cascade	GO:0070371
	Gene expression	GO:0010467
	Glucocorticoid metabolic process	GO:0007186
	Hydrogen peroxide catabolic process	GO:0042744
	Immune system process	GO:0002376
	Lipid metabolic process	GO:0006629
	Lipoprotein catabolic process	GO:0042159
	Negative regulation of cellular protein metabolic process	GO:0032269
	Negative regulation of gene expression	GO:0010629
	Negative regulation of hydrolase activity	GO:0051346
	Negative regulation of MAP kinase activity	GO:0043407
	Peptidyl-methionine modification	GO:0018206
2	**Phosphatidylcholine metabolic process**	**GO:0046470**
	Phospholipid metabolic process	GO:0006644
	Positive regulation of cell death	GO:0010942
	Positive regulation of cholesterol esterification	GO:0010873
	Positive regulation of ERK1 and ERK2 cascade	GO:0070374
	Positive regulation of lipoprotein lipase activity	GO:0051006
	Positive regulation of low-density lipoprotein particle receptor catabolic process	GO:0032805
	Positive regulation of membrane protein ectodomain proteolysis	GO:0051044
	Positive regulation of nitric oxide biosynthetic process	GO:0045429
	Positive regulation of nitric-oxide synthase activity	GO:0051000
	Positive regulation of transcription, DNA-templated	GO:0045893
	Post-translational protein modification	GO:0043687
	Protein oxidation	GO:0018158
	Purine nucleobase metabolic process	GO:0006144
3	**Regulation of amyloid precursor protein catabolic process**	**GO:1902991**
	Regulation of gene expression	GO:0010629
	Regulation of lipid metabolic process	GO:0019216
4	**Regulation of neuron death**	**GO:1901214**
	Regulation of plasma lipoprotein particle levels	GO:0097006

	Regulation of proteasomal protein catabolic process	GO:0061136
	Regulation of protein metabolic process	GO:0051246
	Regulation of protein phosphorylation	GO:0001932
**5**	**Regulation of tau-protein kinase activity**	**GO:1902947**
	Regulation of transcription by RNA polymerase II	GO:0006357
	Response to estrogen	GO:0043627
	Retinoid metabolic process	GO:0001523
	Steroid metabolic process	GO:0008202
	Triglyceride metabolic process	GO:0006641
	Zymogen activation	GO:0031638
	**Macromolecular transport**	
	Bicarbonate transport	GO:0015701
	Lipid transport	GO:0006869
	Lipid transport involved in lipid storage	GO:0010877
	Maintenance of location in cell	GO:0051651
	Negative regulation of cholesterol efflux	GO:0090370
	Negative regulation of protein secretion	GO:0050709
	Nitric oxide transport	GO:0030185
**6**	**NMDA glutamate receptor clustering**	**GO:0097114**
	Oxygen transport	GO:0015671
	Platelet degranulation	GO:0002576
	Positive regulation of endocytosis	GO:0045807
**7**	**Positive regulation of lipid transport across blood-brain barrier**	**GO:1903002**
	Positive regulation of phospholipid efflux	GO:1902995
	Protein import	GO:0017038
	Receptor-mediated endocytosis	GO:0006898
	Thyroid hormone transport	GO:0070327
	Transmembrane transport	GO:0055085
	Vitamin transport	GO:0051180
	**Development and detoxification**	
	Adrenal gland development	GO:0030325
	Animal organ regeneration	GO:0031100
	Artery morphogenesis	GO:0048844
	Blood coagulation	GO:0030195
	Cellular oxidant detoxification	GO:0098869
	Circulatory system development	GO:0072359
	Locomotory exploration behavior	GO:0035641
**8**	**Long-term memory**	**GO:0007616**
	Negative regulation of heterotypic cell-cell adhesion	GO:0034115

**9**	**Neuron projection development**	**GO:0010977**
**10**	**Positive regulation of dendritic spine development**	**GO:0060999**
	Positive regulation of substrate adhesion-dependent cell spreading	GO:1900026
**11**	**Regulation of beta-amyloid clearance**	**GO:1900221**
	Response to drug	GO:0042493
	Response to nutrient	GO:0007584
	**Signal transduction**	
	cGMP-mediated signaling	GO:0019934
	G protein-coupled receptor signaling pathway	GO:0007186
	Integrin-mediated signaling pathway	GO:0007229
	Negative regulation of canonical Wnt signaling pathway	GO:0090090
	Negative regulation of cytokine production involved in immune response	GO:0002719
	Negative regulation of endothelial cell proliferation	GO:0001937
	Negative regulation of interleukin-1 beta production	GO:0032691
	Negative regulation of response to cytokine stimulus	GO:0060761
	Nitric oxide mediated signal transduction	GO:0007263
	Positive regulation of Rho protein signal transduction	GO:0035025
	Regulation of Cdc42 protein signal transduction	GO:0032489
	Signal transduction	GO:0007165
**12**	**Synaptic transmission, cholinergic**	**GO:0007271**
	Transforming growth factor beta receptor signaling pathway	GO:0007179
	**Biomolecular responses and related processes**	
	Acute inflammatory response	GO:0002526
	Acute-phase response	GO:0006953
	Defense response	GO:0006952
	Defense response to bacterium	GO:0042742
	Negative chemotaxis	GO:0050919
	Negative regulation of inflammatory response	GO:0050728
**13**	**Peripheral nervous system axon regeneration**	**GO:0014012**
**14**	**Regulation of behavioral fear response**	**GO:2000822**
	Regulation of innate immune response	GO:0045088
	Response to caloric restriction	GO:0061771
	Response to excess	GO:0002021
	Response to reactive oxygen species	GO:0000302
	Response to oxidative stress	GO:0006979
	**Macromolecular assembly and organization**	
	Chylomicron assembly	GO:0034378

	Chylomicron remodeling	GO:0034371
	Cytoskeleton organization	GO:0007010
	Extracellular matrix organization	GO:0030198
**15**	**Negative regulation of long-term synaptic potentiation**	**GO:1900272**
**16**	**Positive regulation of dendritic spine maintenance**	**GO:1902952**
**17**	**Positive regulation of neurofibrillary tangle assembly**	**GO:1902998**
	Positive regulation of stress fiber assembly	GO:0051496
**18**	**Regulation of neuronal synaptic plasticity**	**GO:0048168**
	Regulation of protein-containing complex assembly	GO:0043254
	Renal absorption	GO:0070293
	Very-low-density lipoprotein particle clearance	GO:0034447
	Virion assembly	GO:0019068
	**Homeostasis**	
	Cellular calcium ion homeostasis	GO:0006874
	Lipid homeostasis	GO:0055088
	Phospholipid homeostasis	GO:0055091
	Protein stabilization	GO:0050821
	Regulation of blood pressure	GO:0008217
**19**	**Regulation of synapse organization**	**GO:0050807**
	Vasodilation	GO:0042311

Numbers of GO terms with neurological implications match those reported in Supplementary Fig. S1.
