# Supplementary figures and images for: Nusinersen Modulates Proteomics Profiles of Cerebrospinal Fluid in Spinal Muscular Atrophy Type 1 Patients
Source: Int J Mol Sci. 2021 Apr 21;22(9):4329. doi: 10.3390/ijms22094329 (PMC8122268; doi:10.3390/ijms22094329)

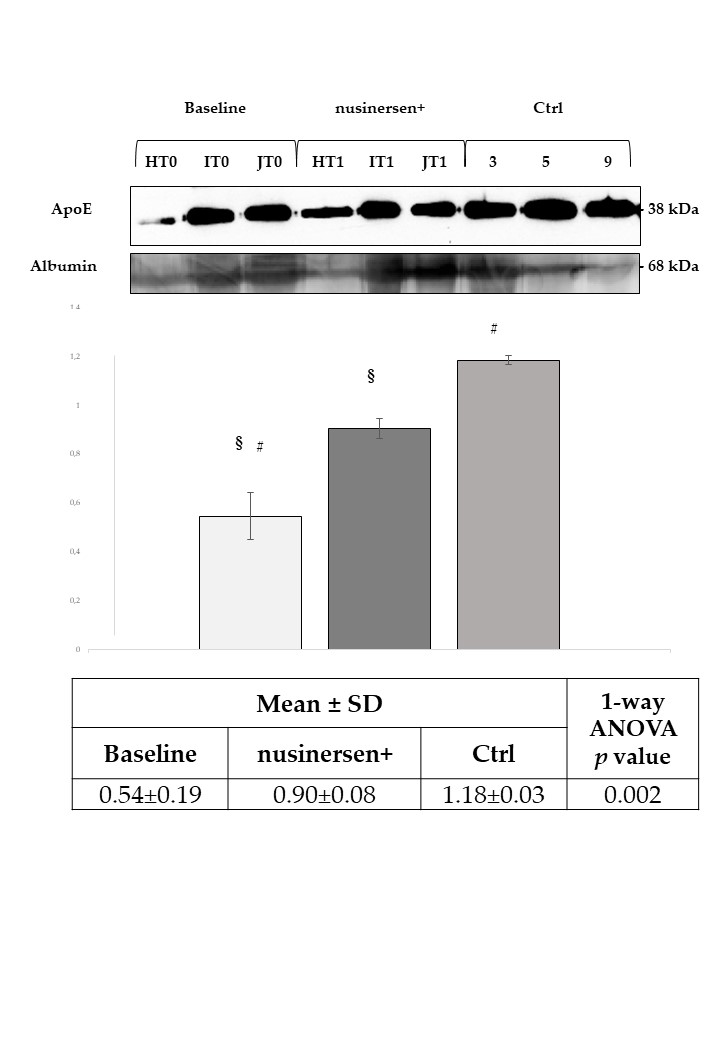

Supplement: Supplementary file 1 [file ijms-22-04329-s001.zip › Supplementary-FIGURE_S2 .JPG]

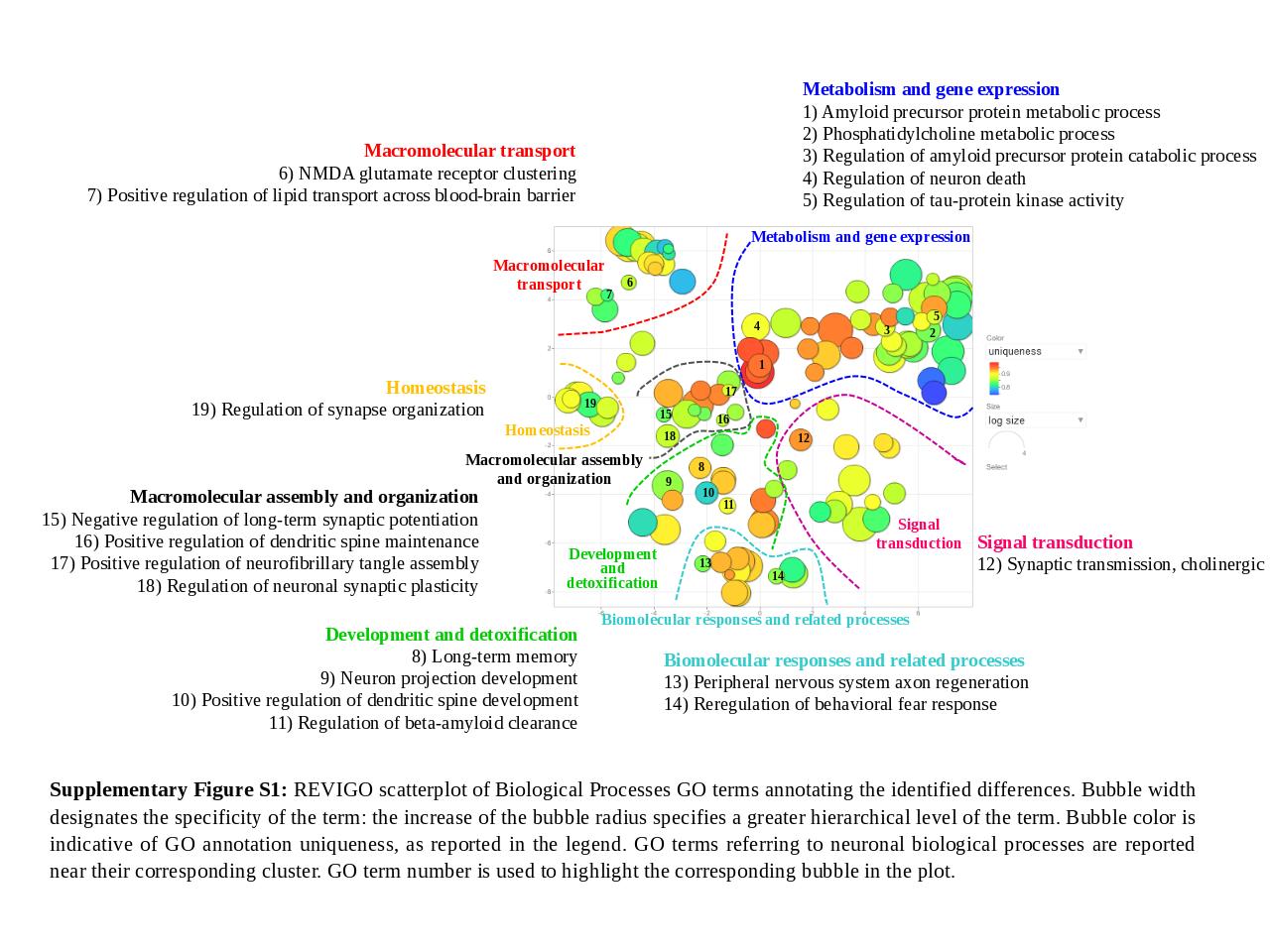

Supplement: Supplementary file 1 [file ijms-22-04329-s001.zip › Supplementary_FIGURE_S1.jpeg]
